# Functional Screening of Hydrolytic Activities Reveals an Extremely Thermostable Cellulase from a Deep-Sea Archaeon

**DOI:** 10.3389/fbioe.2015.00095

**Published:** 2015-07-01

**Authors:** Benedikt Leis, Simon Heinze, Angel Angelov, Vu Thuy Trang Pham, Andrea Thürmer, Mohamed Jebbar, Peter N. Golyshin, Wolfgang R. Streit, Rolf Daniel, Wolfgang Liebl

**Affiliations:** ^1^Department of Microbiology, School of Life Sciences Weihenstephan, Technische Universität München, Freising-Weihenstephan, Germany; ^2^Göttingen Genomics Laboratory, Department of Genomic and Applied Microbiology, Georg-August University Göttingen, Göttingen, Germany; ^3^Laboratoire de Microbiologie des Environnements Extrêmes-UMR 6197 (CNRS-Ifremer-UBO), Institut Universitaire Européen de la Mer, Université de Bretagne Occidentale, Plouzané, France; ^4^School of Biological Sciences, Bangor University, Bangor, UK; ^5^Fakultät für Mathematik, Informatik und Naturwissenschaften Biologie, Biozentrum Klein Flottbek, Universität Hamburg, Hamburg, Germany

**Keywords:** functional screenings, extreme thermostable protein, archaeal endoglucanase, enzymatic characterization

## Abstract

Extreme habitats serve as a source of enzymes that are active under extreme conditions and are candidates for industrial applications. In this work, six large-insert mixed genomic libraries were screened for hydrolase activities in a broad temperature range (8–70°C). Among a variety of hydrolytic activities, one fosmid clone, derived from a library of pooled isolates of hyperthermophilic archaea from deep sea vents, displayed hydrolytic activity on carboxymethyl cellulose substrate plates at 70°C but not at lower temperatures. Sequence analysis of the fosmid insert revealed a gene encoding a novel glycoside hydrolase family 12 (GHF12) endo-1,4-β-glucanase, termed Cel12E. The enzyme shares 45% sequence identity with a protein from the archaeon *Thermococcus sp*. AM4 and displays a unique multidomain architecture. Biochemical characterization of Cel12E revealed a remarkably thermostable protein, which appears to be of archaeal origin. The enzyme displayed maximum activity at 92°C and was active on a variety of linear 1,4-β-glucans like carboxymethyl cellulose, β-glucan, lichenan, and phosphoric acid swollen cellulose. The protein is able to bind to various insoluble β-glucans. Product pattern analysis indicated that Cel12E is an endo-cleaving β-glucanase. Cel12E expands the toolbox of hyperthermostable archaeal cellulases with biotechnological potential.

## Introduction

The search for novel enzymes for biotechnological and pharmaceutical applications must focus on proteins displaying the desired properties under process-relevant conditions, which are often harsh, especially with regard to salt or solvent concentrations, pH, and temperature. In this regard, process conditions resemble the conditions in extreme environments. Microorganisms inhabit a multitude of such environments, characterized by high temperature, salinity, or extreme pH (Grant et al., 2004; Li et al., 2014). Accordingly, extreme habitats represent a good source for enzymes active under extreme conditions (Simon et al., 2009; Delavat et al., 2012). Two distinct strategies are available for the identification of enzymes from extreme environments. While metagenomics enable the exploration of phylogenetic and biochemical characteristics of microbial consortia without the need for cultivation (Handelsman et al., 1998), culture-based approaches overcome restrictions of metagenomic screening.

The choice of an appropriate sampling environment is generally important when screening for certain enzymatic activities (Taupp et al., 2011) but interesting enzymes have also been discovered in samples from environments in which the respective functions were initially not expected or observed (Voget et al., 2003; Delavat et al., 2012). Therefore, it has been suggested to broaden functional screenings, for example, to include more than one specific enzymatic activity (Leis et al., 2013). Although sequence similarity and functional screenings are both well established, functional screens appear to be better suited for the discovery of completely new enzymatic functions encoded by the genetic material (Langer et al., 2006; Liebl et al., 2014).

In this study, we performed a functional screening of fosmid expression libraries in order to identify hydrolytic activities for biotechnological applications. The fosmid inserts were derived from mixed genomes originating from mesophilic and thermophilic bacteria as well as hyperthermophilic archaeal (HA) isolates (deep sea environments), enrichment cultures from ship worm digestive tracts, and uncultured microorganisms (river sediment, elephant feces). The screening was performed at different temperatures, ranging from 8 to 70°C. The application of the same expanded temperature range for all functional screenings, independent of the nature of the source of the genomic DNA, increased the number of unique identified proteins. By this route, we were able to identify a total of 60 different activities (esterases, lipases, and glycoside hydrolases). One cellulolytic clone from a library of uncharacterized archaeal isolates cultivated from hydrothermal vents was of particular interest due to its activity at elevated temperatures. Sequence analysis and biochemical characterization revealed an extremely thermostable endoglucanase, termed Cel12E, which was able to hydrolyze a variety of β-1,4-linked polysaccharides. The high thermostability and broad substrate specificity of Cel12E make this enzyme a promising candidate for industrial degradation of lignocellulosic biomass.

## Materials and Methods

### Bacterial strains and vectors

*Escherichia coli* EPI300-T1 (Epicentre, Madison, USA) was used for screening the genomic libraries cloned in pCC1FOS large insert fosmids (Epicentre). The *E. coli* strains XL1-Blue (Stratagene, La Jolla, USA) and DH10B (Invitrogen, Carlsbad, USA) were used for transformation and propagation of recombinant plasmids. *E. coli* strain BL21(DE3) was used as the host for pET21a(+) expression vector. *E. coli* was grown and maintained on LB media with appropriate antibiotic supplementation with ampicillin (100 μg/ml) and chloramphenicol (12.5 μg/ml) at 37°C overnight.

### Generation of genomic DNA libraries and activity-based screenings

Three metagenomic DNA libraries were available for functional screenings: low-to-mid temperature libraries were derived from sediments from the river Elbe (Rabausch et al., 2013), elephant feces and ship worm enrichment cultures on CMC (Ilmberger et al., 2012), each containing 960 fosmid clones. Three further fosmid libraries contained inserts of mixed genomic DNA of enrichment cultures from deep sea hydrothermal vents. A total of 788 uncharacterized, individually grown strains were subdivided into three physiological groups (251 thermophilic bacteria, 194 mesophilic bacteria, and 343 hyperthermophilc archaea) and used to prepare these “mixed genome” libraries. Each library represented one of the three physiological groups. Cells from 20 to 30 ml culture volume were harvested by centrifugation at 8,000 × rpm at 4°C for 5 min. For cell lysis, 100 μl 10% sarkosyl, 100 μl 10% SDS, and 50 μl proteinase K (20 mg/ml stock) were added and mixed gently. The lysis reaction was incubated for 1 h at 55°C and slowly stirred several times. RNase A was added (20 μl of 50 μg/ml stock) and incubated for 20–30 min at 37°C. After cell lysis, the genomic DNA of each strain was isolated. The best DNA yield and quality were obtained with the standard phenol-chloroform method according to Sambrook et al. (1989). To obtain the three “mixed genome” DNA libraries, the DNA extracts of all isolates belonging to the same physiological group were mixed using equal DNA amounts and subjected to further cloning into pCC1FOS fosmids (according to the Epicentre manufacturer instructions). The isolated microorganisms are deposited as -80°C DMSO-stocks in the UBO Culture Collection UBOCC^1^ of the laboratory of Microbiology of Extreme Environments at IUEM-UBO (Plouzané, France).

*Escherichia coli* EPI300-T1 transformed with the fosmid library was grown on LB agar plates at 37°C overnight to yield single colonies. Next, replica plating was used to transfer the library colonies on LB agar indicator plates containing Fosmid Autoinduction Solution (Epicentre, Madison, USA) for activation of the *oriV* origin and different substrates: 1.0% (v/v) tributyrin, 1.0% (v/v) triolein supplemented with rhodamine B [according to Kouker and Jaeger (1987)], 0.1% (w/v) xylan (from oat spelts), 0.1% (w/v) carboxymethyl cellulose (CMC, sodium salt, low viscosity), 0.3% (w/v) starch (soluble). All substrates were purchased from Sigma-Aldrich (Germany). The original LB agar plates were stored at 4°C to enable the identification of positive clones after the functional screenings. After growth at 37°C overnight, the replicated colonies on the LB agar indicator plates were subjected to functional screenings for 1 week at temperatures from 8 to 20°C or for 2–3 days when incubating at temperatures from 30 to 70°C. Agar plate screening for (hemi-) cellulose degradation was monitored using Congo red staining solution of 0.1% (w/v) followed by repeated washing with 1.0M sodium chloride solution (Wood et al., 1988). Halo formation around the colonies indicated degradation of substrates due to hydrolytic activity. Functional screening in microtiter plates was performed with Cibacron red dyed substrates (xylan and CMC) according to Ten et al. (2005). Starch degradation was visualized by the addition of Lugol’s iodine solution (0.33% w/v elemental iodine and 0.66% w/v potassium iodide). DNA from positive fosmid clones was isolated and re-transformed in *E. coli* EPI300 cells to confirm the observed activity. Cloning of fosmid fragments was performed with pCR^®^2.1-XL-TOPO^®^ (Invitrogen, USA) for confirmation of the phenotype and for sequencing purposes.

### Sequence analysis

Complete sequencing of selected DNA inserts was performed in the Göttingen Genomics Laboratory (G_2_L) using a combination of Sanger and 454-pyrosequencing technology. The generation of 454 shotgun libraries was performed following the manufacturer instructions (Roche, 454 Life Sciences, Branford). Libraries were sequenced using the FLX Titanium chemistry and 25,591 single reads were generated. The shotgun reads were assembled *de novo* with Newbler Assembler V2.6 (Roche, Branford), resulting in 153 contigs (>500 bp). Fosmid mapping was achieved by sequencing from both fosmid ends using oligonucleotide primers abi-for (5′-ACGACGTTGTAAAACGACGGCCAG-3′) and abi-rev (5′-TTCACACAGGAAACAGCTATGACC-3′) with Sanger technology. ORF prediction and annotation was performed with SEED (Overbeek et al., 2005), SignalP (Petersen et al., 2011) was used for the prediction of signal peptides. BlastP (Altschul et al., 1990) was used for sequence similarity search against the *nr* database, conserved protein domains were searched in the Pfam database version 27.0 (Punta et al., 2012). The prediction of domain linkers was performed with the DLP-SVM web service (Ebina et al., 2009).

### Construction of expression vectors

Gene *cel12E* of fosmid HA-cmc-1 was amplified by PCR with Phusion F530S DNA polymerase (ThermoFisher Scientific, Waltham, MA, USA) according to the manufacturer’s instructions. The PCR product of *cel12E* was cloned in pET21a(+) expression vector using Gibson Assembly™ (New England Biolabs, Ipswich, MA, USA). Amplicons obtained with primers SP-HACMC-F (5′- TTTAAGAAGGAGATATACAATGAAAAGCATTGCACTTG -3′) and HACMC-R (5′-GTGGTGCTCGAGTGCGGCCTCACTGTGGCGTCCAGATA-3′) were full-length Cel12E with the predicted signal peptide encoded at the N-terminus. A truncated version of Cel12E omitting the signal peptide sequence was obtained with HACMC-F (5′-TTTAAGAAGGAGATATACAATGCAGGAGACAACAGTGCTGGA-3′) and HACMC-R.

### Expression and purification of recombinant cel12E

*Escherichia coli* cultures (500 ml) were grown in Erlenmeyer flasks to mid-log phase (absorbance at 600 nm wavelength ranging from 0.6 to 0.7) and induced with isopropyl-β-d-thiogalactopyranoside (IPTG) at a final concentration of 1.0 mM. After 4 h at 37°C, the cultures were harvested by centrifugation (5,000 × *g* for 5 min) and disrupted by French pressure cell (SLM Aminco, Urbana, IL, USA). EDTA-free Protease Inhibitor Cocktail Tablets (Roche Diagnostics, Mannheim, Germany) were used during protein purification to prevent degradation of the target protein. After removal of the cell debris by centrifugation (21,000 × *g* for 15 min), the lysate was subjected to heat treatment (80°C for 20 min). The supernatant containing the thermostable and soluble protein of interest was purified using an Äkta Explorer fast protein liquid chromatography system (GE Healthcare, Little Chalfont, UK) with a SOURCE™ 15Q anion exchange column in 50 mM Tris-HCl buffer, pH 8.0 (buffer A) and sodium chloride gradient (buffer B, 50 mM Tris-HCl, pH 8.0, 1.0 M NaCl). Gel filtration on Superdex 200 column for Cel12E was performed in buffer A supplemented with 150 mM NaCl. Protein separation and purity were determined with sodium dodecyl sulfate polyacrylamide gel electrophoresis (SDS-PAGE) based on Laemmli (1970).

### Enzyme assays

Microcrystalline cellulose (Avicel PH-101), CMC (low viscosity), hydroxyethyl cellulose (HEC), xylan (from oat spelts, birchwood, and larchwood), chitin, chitosan, lichenan (from *Cetraria islandica*), laminarin (from *Laminaria digitata*), and starch (from potato) were purchased from Sigma (St. Louis, MO, USA); β-glucan (from barley), pachyman (from *Poria cocos*), xyloglucan (from tamarind), arabinoxylan (medium viscosity and insoluble form, from wheat), arabinan (from sugar beet), arabinogalactan (from larch wood), galactan and pectic galactan (from potato and lupine), glucomannan (from konjac), galactomannan (from guar), and mannan (from ivory nut) were obtained from Megazyme (Wicklow, Ireland). Phosphoric acid swollen cellulose (PASC) was prepared from Avicel (Wood, 1988). Activity of recombinant proteins was determined using the 3,5-dinitrosalicylic acid (DNS) colorimetric assay (Miller, 1959). One unit of enzymatic activity was defined as the amount of enzyme which liberates 1.0 μmol of reducing sugar ends per minute from the substrate. The standard enzyme activity assay was performed with 0.1 μg/ml of Cel12E in 50 mM MES buffer 2-(N-morpholino)ethanesulfonic acid, pH 5.5 at 92°C in an oil bath rotary shaker (Infors HT Aquatron, Bottmingen, Switzerland). The pH optimum for activity was determined using different buffers (each at 50 mM): glycin-HCl (pH 2–3), sodium acetate buffer (pH 4–6), and phosphate buffer (pH 5.5–8). For enzyme thermoinactivation kinetics, 0.5 μg/ml of Cel12E were incubated over various time intervals at 80, 92, and 97°C. Residual enzymatic activity was assessed using 0.1 μg/ml of Cel12E in 50 mM MES buffer (pH 5.5) on 1.4% (w/v) CMC.

### Analysis of polysaccharide degradation products

The degradation products of enzymatic hydrolysis of PASC and cellodextrins were analyzed using thin layer chromatography (TLC). Two microliters of samples with cellodextrins (from cellobiose to celloheptaose) and 22.5 μl of samples with PASC were spotted on silica gel type 60 F254 aluminium plates (Merck, Darmstadt, Germany). The mobile phase consisted of acetonitrile/water (80:20 v/v). The separation was carried out twice (with thorough drying of the plates between consecutive separation steps to remove any residual mobile phase), before the degradation products were visualized by spraying the plates with a freshly prepared mixture of 10 ml stock solution (1 g diphenylamine and 1 ml aniline dissolved in 100 ml acetone) and 1 ml 85% phosphoric acid followed by incubating the plates at 120°C for 15 min.

### Substrate binding assays

Insoluble polysaccharides (0.5% final concentration) were mixed with 1.5 μg/μL purified Cel12E in 50 mM MES buffer containing 200 mM NaCl and incubated overnight at 6°C in a Thermomixer Comfort (Eppendorf, Germany). Protein bound on the insoluble substrate was centrifuged (21,000 × *g* for 30 min) and washed four times; bound proteins were then eluted with addition of protein loading buffer containing SDS and loaded on SDS-PAGE gels.

## Results

### Screening of metagenomic libraries for hydrolytic activities

**Figure 1:**
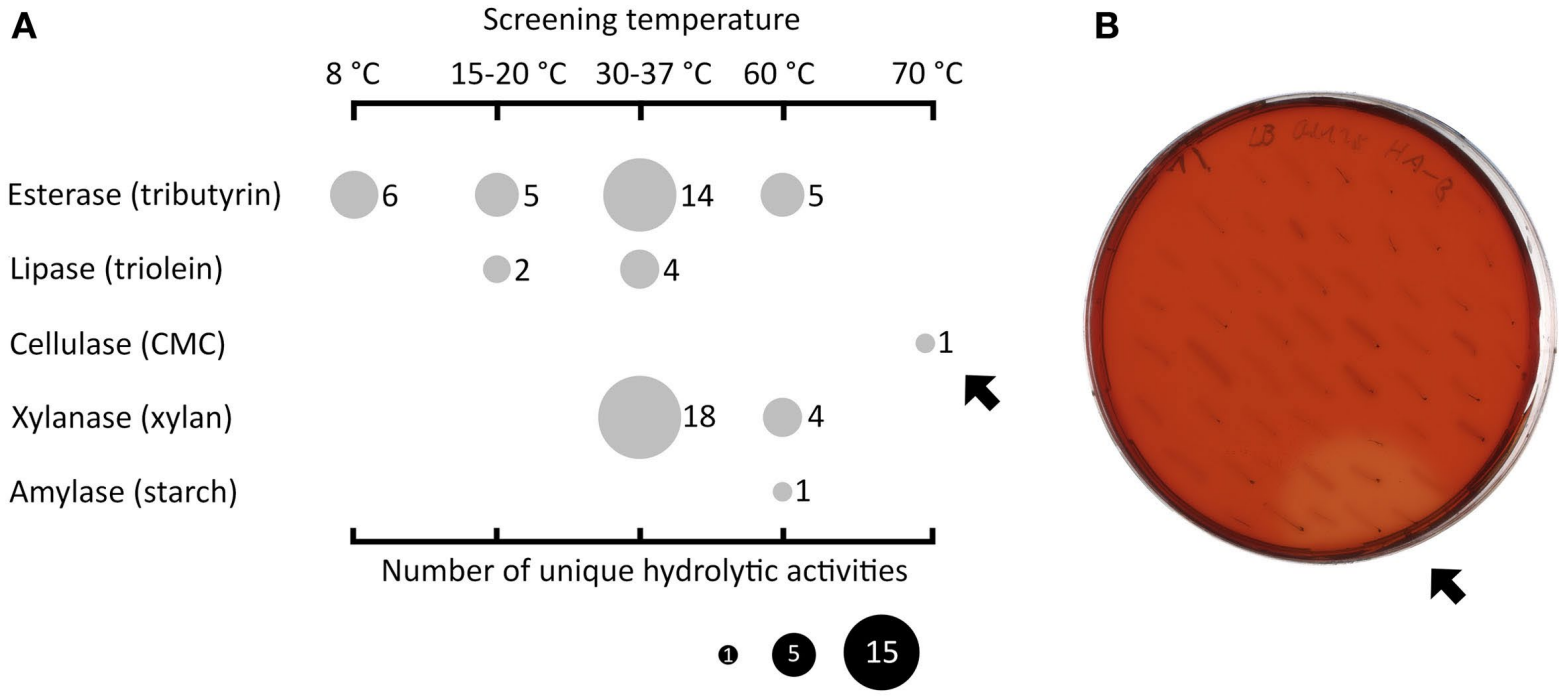
**Summary of functional screenings of diverse fosmid libraries in *E. coli***. Esterase activity was determined by the presence of clear hydrolysis halos around the colonies on LB agar indicator plates containing tributyrin (1% v/v). Lipase-active clones gave a fluorescent halo on plates with trioleine (1% v/v) supplemented with Rhodamine B when exposed to UV light (Kouker and Jaeger, 1987). (Hemi-)cellulolytic activities were visible by release of Cibacron red from dyed insoluble substrates or clear hydrolysis halos on LB substrate indicator plates containing 0.1% (w/v) carboxymethyl cellulose or oat spelt xylan. Amylolytic activity was visualized by staining the 0.3% (w/v) starch plates with Lugol’s iodine solution. **(A)** Overview of all hydrolytic activities (vertical axis) identified at different incubation temperatures (horizontal axis). The size of the filled circles indicates the number of unique clones in dependence of the substrate and screening temperatures used. The black arrows indicate one particular fosmid clone termed HA-cmc-1 that was active on CMC at 70°C after 2 days of incubation **(B)**.

Fosmid libraries containing genomic DNA derived from different habitats were functionally screened for hydrolytic activities in the mesophilic screening host *E. coli*. In order to reflect the conditions of the respective sources of the libraries, the functional screening was performed at four different temperature ranges (8°C, 15–20°C, 30–37°C, and 60–70°C). Figure 1A displays the types and number of discovered activities depending on the screening temperatures. The highest number of active fosmid clones was found in the mesophilic range (n = 36). Below 30°C, 13 positive clones were detected and above 37°C, 11 positive clones were found. In particular, one fosmid clone (named HA-cmc-1) was only active at 70°C, the highest temperature used in the screening. When incubated at 70°C for 2 days, colonies of *E. coli* carrying HA-cmc-1 showed a clear hydrolysis halo after Congo red staining when grown on agar plates containing CMC (Figure 1B). This indicated the presence of a thermophilic cellulase encoded by the fosmid insert. This fosmid contained a DNA insert derived from HA enrichment cultures, which originated from deep sea hydrothermal vents.

### Sequencing and bioinformatic analysis of fosmid HA-cmc-1

**Figure 2:**
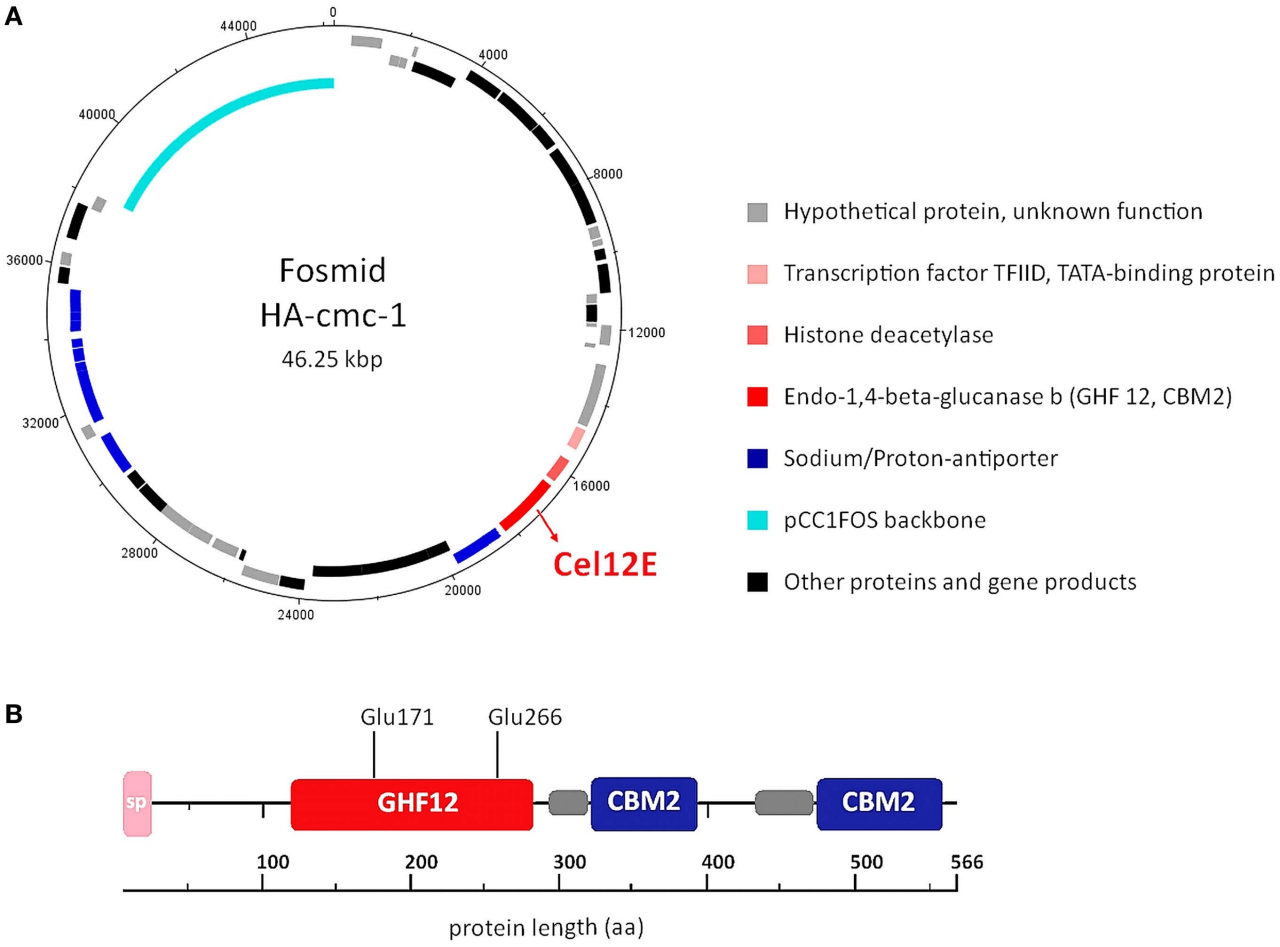
**DNA and amino acid sequence analysis of the cellulase active-fosmid HA-cmc-1 and of the Cel12E protein**. **(A)** Predicted ORFs in forward direction (outermost ring) and reverse direction (second ring). Possible biological functions are depicted in different colors. **(B)** Multidomain architecture of Cel12E. A signal peptide (24 amino acids), the catalytic GHF12 domain, and two carbohydrate binding modules (CBM2) at the C-terminus were predicted. The predicted catalytic nucleophile Glu-171 and acid-base residue Glu-266 are indicated. Possible domain linker regions that could be predicted are shown as gray boxes.

In order to reveal the gene(s) carried on the cellulase activity-encoding fosmid HA-cmc-1, the fosmid was sequenced using a combination of Sanger and 454-sequencing. Assembly of the obtained sequence reads resulted in a fosmid insert of 38,175 bp DNA (Genbank/EMBL/DDBJ accession no. LN850140). Sequence analysis revealed 48 putative open reading frames (ORFs) that are represented in the fosmid map on Figure 2A. All predicted ORFs encoded proteins with highest similarity to *Thermococcus* species. Inspection of the predicted ORFs for the presence of glycoside hydrolase domains revealed that ORF 23 encodes a predicted glycoside hydrolase family 12 (GHF12, pfam family pf01670) protein of 566 amino acids with a predicted molecular mass of 62.3 kDa and an isoelectric point of 4.26. The protein showed amino acid sequence similarity to endo-1,4-β-glucanase b of *Thermococcus sp*. AM4 (Genbank accession no. YP_002581913.2, 45% identity, blastp E-value of 2 × e^-82^). Analysis of the domain structure of this ORF revealed one GH12 module followed by two separated carbohydrate binding modules (CBMS) of family 2 (CBM2) (Figure 2B). Multiple sequence alignments revealed two conserved glutamates as catalytic residues in the active site (nucleophile Glu-171 and the acid-base Glu-266). CBM2 members are known to bind cellulose, chitin, and xylan^2^ (Lombard et al., 2014). A signal peptide sequence motif could be predicted with a suggested signal peptidase cleavage between the residues Ala-24 and Gln-25.

### Expression and purification of cel12E

**Figure 3:**
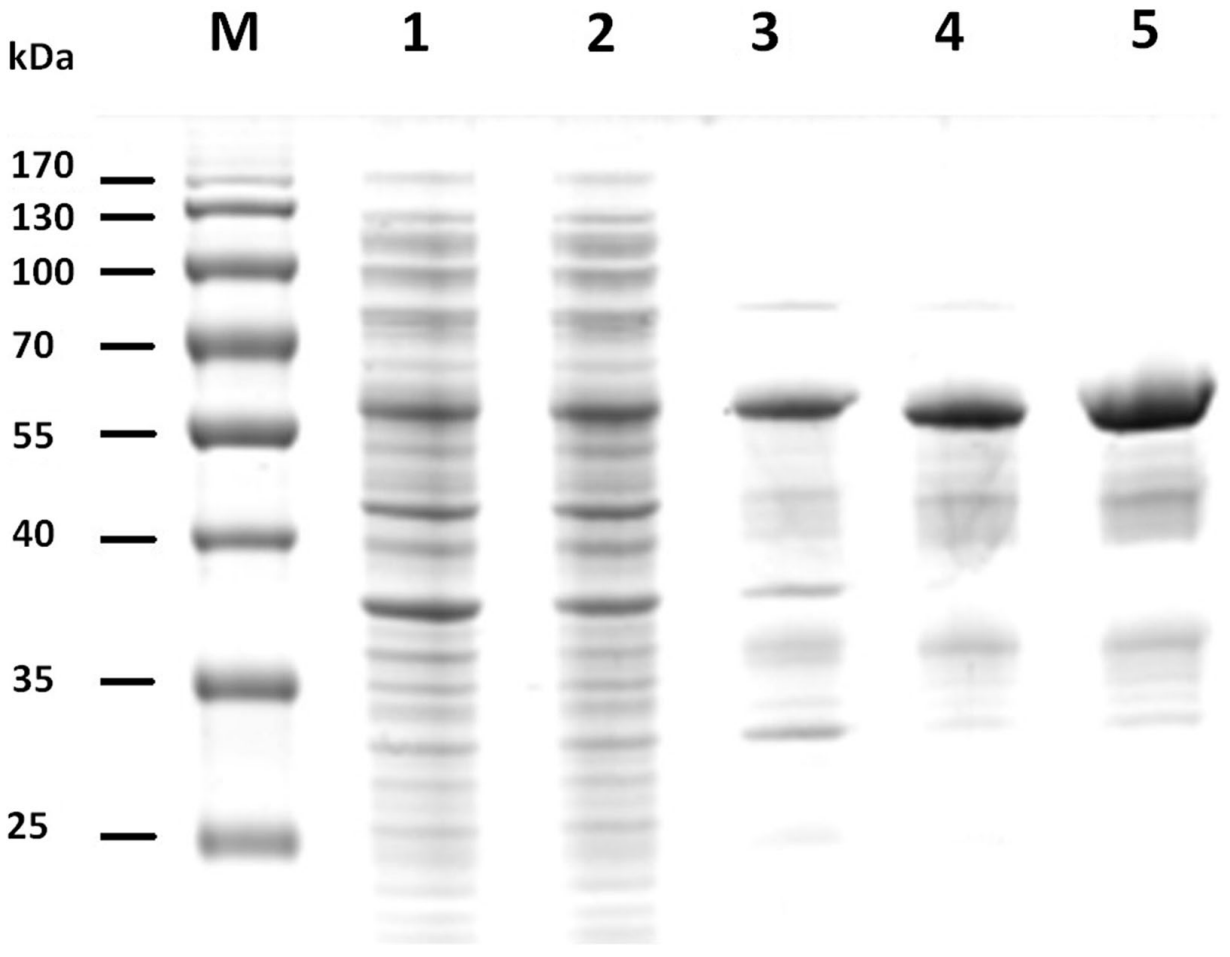
**SDS-PAGE of fractions of recombinant Cel12E throughout the purification steps**. The figure shows the untreated raw extract of *E. coli* BL21 after protein expression (lane 1), the soluble fraction (lane 2), heat-treated supernatant (lane 3), collected fractions after SOURCE 15Q anion exchange (lane 4), and Superdex 200 gel filtration chromatography (lane 5). M, molecular size marker.

The gene sequences encoding the entire preprotein and a N-terminally truncated form lacking the predicted signal peptide were cloned into the pET21a(+) expression vector without the addition of any purification tag sequences. Although both enzyme variants were functionally expressed in *E. coli* BL21(DE3), the N-terminally truncated form (termed Cel12E) yielded 2.3 times higher functional protein production levels. Cel12E displayed hydrolytic activity toward CMC at temperatures above 80°C. Due to the inherent thermostable nature of the target protein, no affinity tag was needed for purification purposes. Protein purification was achieved by heat treatment of *E. coli* proteins at 80°C, followed by anion exchange chromatography and size exclusion chromatography (purification details are provided in Table 1). SDS-PAGE analysis (Figure 3) revealed a band corresponding to the expected 59.96 kDa molecular mass. Subsequently, the expressed and purified target protein was characterized biochemically.

**Table 1:** **Purification table for Cel12E**.

Fraction of purification	Volume (ml)	Total protein (mg)	Total activity (U) **×** 10^3^	Specific CMCase activity (U/mg)	Yield (%)	Fold purification
Crude cell extract	27.5	361.1 ± 3.9	14.2 ± 2.6	39.3 ± 7.5	100 ± 18.6	1.0 ± 0.2
Heat treated extract	22.5	60.3 ± 3.7	9.8 ± 0.9	162.3 ± 9.5	68.9 ± 6.1	4.1 ± 0.2
Anion exchange chromatography	0.4	17.7 ± 0.1	7.8 ± 0.6	440.2 ± 31.9	53.9 ± 6.0	11.2 ± 0.8
Gel filtration chromatography	1.6	8.0 ± 1.1	5.6 ± 1.1	700.4 ± 75.8	39.6 ± 7.9	17.8 ± 1.9

*Values are average values ± 1 × SD from two independent purifications*.

### Substrate specificity and product pattern

**Figure 4:**
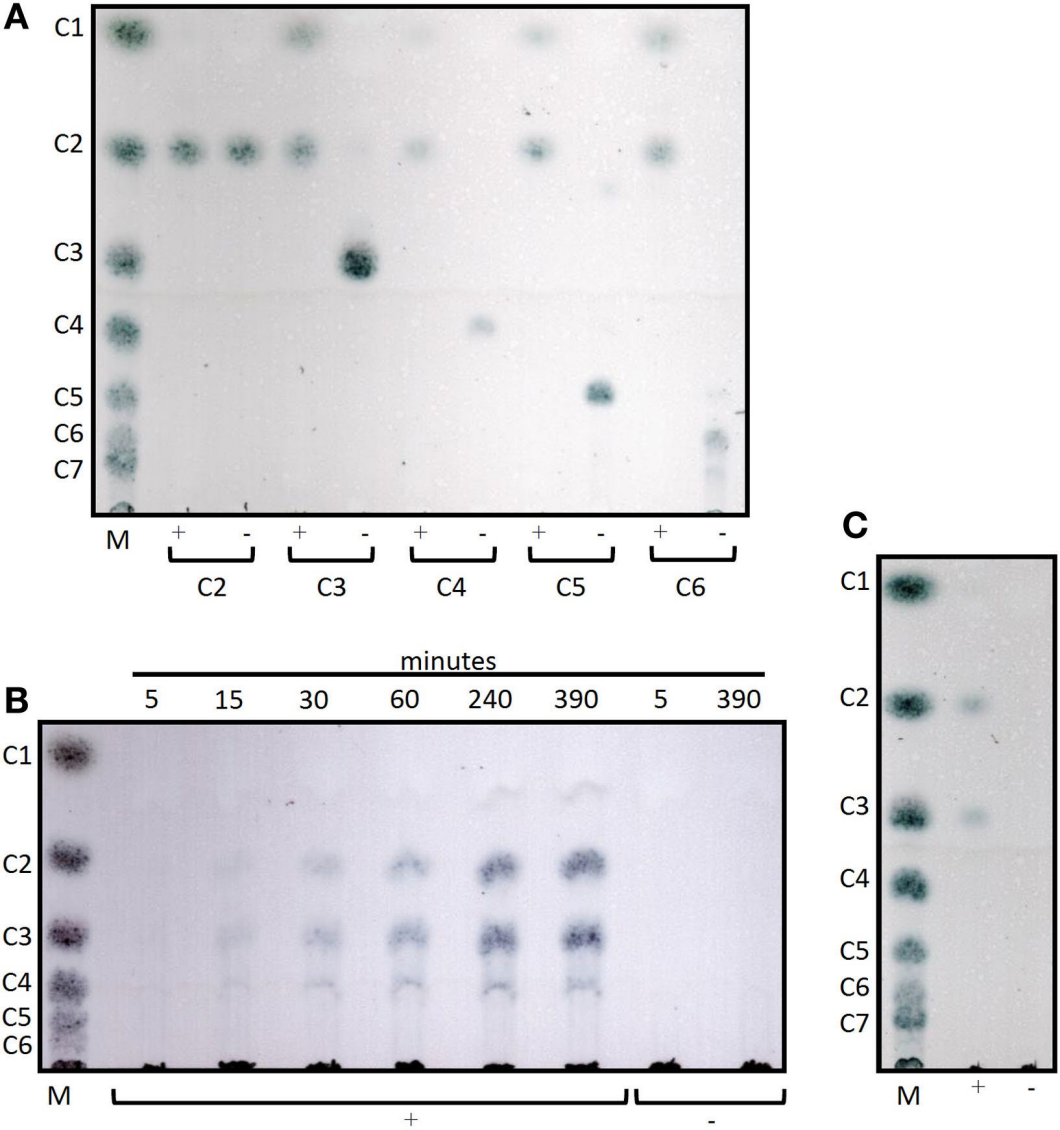
**Thin layer chromatograms of the hydrolysis products of Cel12E**. **(A)** 0.1% (w/v) of cello-oligosaccharides from cellobiose (C2) to cellohexaose (C6) were incubated in 50 mM MES buffer pH 5.5 at 92°C for 6 h, followed by incubation at 60°C for 2 days with Cel12E at 52 μg per ml (+) or without enzyme (-, negative control) Cel12E. Samples were taken at the indicated time points and 2 μL were spotted onto a TLC plate. **(B)** Time course of the hydrolysis of PASC by Cel12E. 0.5% (w/v) PASC was incubated in 50 mM MES buffer pH 5.5 at 80°C with Cel12E at 0.2 μg per ml (+) or without enzyme (-, negative control) Cel12E. Samples were taken at the indicated time points and 22.5 μL were spotted onto a TLC plate. The incubation of the reaction mix was continued for 2 days at 60°C **(C)**. The marker (M) contains cello-oligosaccharides from glucose (DP = 1) to cellohexaose [DP = 6, **(B)**] or celloheptaose [DP = 7, **(A,C)**].

Cel12E showed highest specific activity against β-1,4-glycosidic bonds of various linear glucan polysaccharides like PASC, barley β-glucan, lichenan, and modified soluble cellulose substrates (CMC, hydroxylethyl cellulose). In contrast to this, the activity of Cel12E toward microcrystalline cellulose was relatively low (Table 2). In addition to these cellulosic substrates, Cel12E also displayed side activities toward xyloglucan, glucomannan, and different types of xylans (approximately, 0.1–2.9% relative activity compared with lichenan). In order to determine intermediate and final products of the hydrolysis of PASC and various cellulose oligosaccharides (DP 2: cellobiose to DP 6: cellohexaose), TLC analysis was performed. Cellobiose and cellotriose accumulated as products when PASC was used as substrate at a Cel12E concentration of 0.2 μg per ml, whereas cellotetraose was only visible to a lesser extent. High-molecular weight intermediate products were not visible. Also, no glucose spots were detected. On the other hand, the hydrolysis of soluble cello-oligosaccharides at a high Cel12E concentration (52 μg per ml) showed degradation of cellotriose, cellotetraose, cellopentaose, and cellohexaose to monomeric glucose and cellobiose, showing that the enzyme can liberate glucose from cellotriose and larger cello-oligosaccharides under these conditions. Cellobiose was not hydrolyzed by Cel12E (Figure 4). Furthermore, the binding properties of Cel12E toward insoluble polysaccharides were studied. The enzyme was shown to bind to microcrystalline and amorphous cellulose as well as mixed linkage β-glucan and chitin. Binding of xylan was not observed.

**Table 2:** **Substrate specificity of Cel12E**.

Substrate (% concentration, w/v)	Type(s) of glycosidic linkage and main sugar monomers	Specific activity (U/mg protein **±** SD)	Binding
**POLYSACCHARIDES FROM GLUCOSEMONOMERS**			
Carboxymethyl cellulose (2.0%)	β-1,4 only	692.3 ± 55.7	
β-glucan from Barley (2.0%)	Mixed β-1,3 and β-1,4	317.7 ± 3.8	Yes
Lichenan (0.5%)	Mixed β-1,3 and β-1,4	272.0 ± 6.9	
Hydroxyethyl cellulose (2.0%)	β-1,4 only	107.7 ± 8.8	
PASC (2.0%)	β-1,4 only	33.6 ± 3.8	Yes
Avicel PH-101 (2.0%)	β-1,4 only	0.03 ± 0.01	Yes
Pachyman	β-1,3 only	Not detected	Yes
Laminarin	β-1,3 and β-1,6	Not detected	
Starch	Mixed α-1,4 and α-1,6	Not detected	
**POLYSACCHARIDES FROM VARIOUS SUGAR MONOMERS**			
Xyloglucan from tamarind (0.5%)	β-1,4 glucose backbone, xylose sidechains	2.9 ± 0.2	
Glucomannan from konjac (0.125%)	β-1,4 glucose and mannose backbone galactose sidechains	2.8 ± 0.1	
Arabinoxylan from oat spelts (0.5%)	β-1,4 xylose backbone, arabinose and xylose sidechains	0.38 ± 0.01	No
Arabinoxylan from wheat (0.5%) iinsoluble		0.12 ± 0.02	
Arabinoxylan from wheat (0.5%) medium viscosity		0.10 ± 0.01	
Glucuronoxylan (0.5%) from birch wood (0.5%)	β-1,4 xylose backbone, 4-O-methyl-glucuronic acid sidechains	0.20 ± 0.04	
Chitin	β-1,4 *N*-acetyl-glucosamine backbone	Not detected	Yes
Chitosan	β-1,4 glucosamine and *N*-acetyl-glucosamine backbone	Not detected	No

*Activities were determined at 92°C, pH 5.5. No hydrolytic activity of Cel12E could be detected toward the following polysaccharides (incubation overnight at 60°C, pH 6.0): arabinan (from sugar beet), arabinogalactan (from larch wood), galactans and pectic galactans (from lupine and potato), galactomannan (from guar), and mannan (from ivory nut)*.

### Biochemical characterization of cel12E

**Figure 5:**
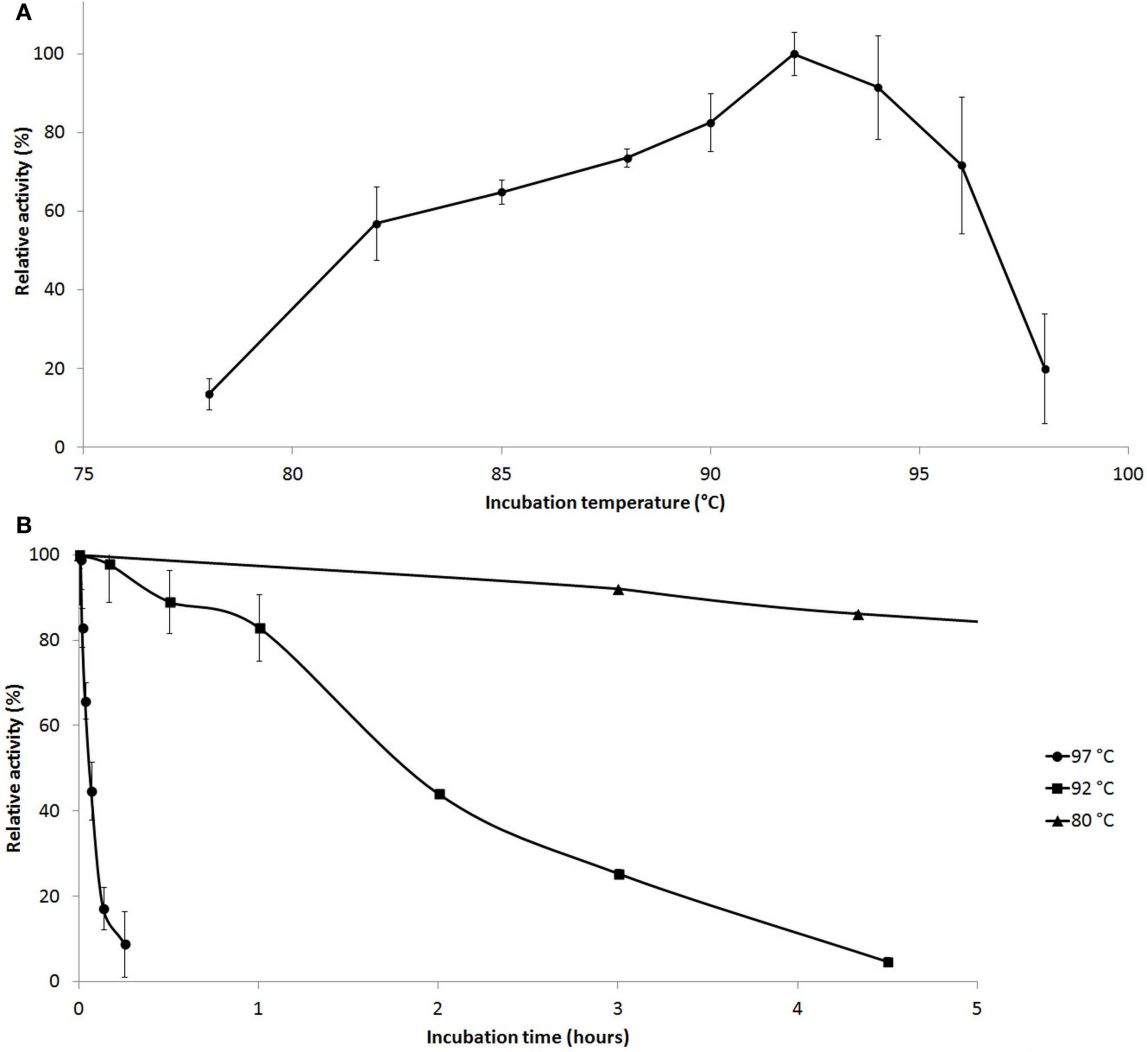
**Effect of temperature on Cel12E activity**. **(A)** Influence of the temperature on the activity of Cel12E toward CMC at pH 5.5 in a 10-min assay. **(B)** Thermal inactivation kinetics of Cel12E at various temperatures. The purified enzyme was incubated at a concentration of 0.5 μg/ml at 80°C (triangles), 92°C (squares), or 97°C (circles) for different periods of time before determining the residual activity at 92°C in 50 mM MES buffer, pH 5.5 with CMC as substrate. Activity data are represented as relative activity from duplicate measurements (±SD).

Enzyme activity measurements were carried out at various temperatures and pH values. Cel12E was found to be a highly thermostable protein that was most active between 90 and 95°C (10 min activity assay at pH 5.5, Figure 5A) with an optimal pH value around 5.5. The enzyme’s half-life of thermoinactivation at 92°C was approximately 2 h, while the enzyme retained more than 80% of its activity even after 4.5 h of incubation at 80°C (Figure 5B). Enzyme kinetics assays, performed with CMC as the substrate, revealed an apparent *V*_max_ value of 1,025 U/mg protein and an apparent *K_m_*-value of 2.35 mg/ml. The influence of several salts and additives on the enzymatic activity was examined at pH 5.5 and 92°C (Table S1 in Supplementary Material). It is noteworthy that the addition of the reducing agent dithiothreitol (DTT) improved enzymatic activity by 65.9–146.2% when added at final concentrations of 10 and 1.0 mM, respectively. An activity increase of about 75% was observed in the presence of 500 mM NaCl or KCl. Interestingly, supplementation with CoCl_2_ and MnCl_2_ at low concentrations led to an increase of the enzymatic activity. The strongest effects were observed with concentrations of 0.5 mM CoCl_2_ (210.9 ± 5.1% relative activity) or 1.0–2.0 mM MnCl_2_ (188.5 ± 22.6% relative activity). Higher concentrations of these bivalent heavy metal ions caused inhibitory effects (data not shown). The promoting effect of these ions at concentrations of 1.0 mM was shown to be selective and reversible by complexation of the ions with excess amounts of EDTA (10 mM) (Table S2 in Supplementary Material). The order of the supplementation of ions and chelating agent did not have an influence on this result. Interestingly, the activating effects of monovalent salts and bivalent metal ions were additive: a combination of 0.4 M NaCl and 0.3 M KCl increased the enzyme’s specific activity by a factor of 1.56, which could be boosted by the addition of 0.2 mM CoCl_2_ to 430% of the control without supplementations (data not shown).

## Discussion

Recent examples of extremely thermostable hydrolases, isolated via functional screenings, include esterases and lipases from hot solfataric springs and compost samples (Rhee et al., 2005; Leis et al., 2015), esterases from hypersaline deep sea brines (Alcaide et al., 2015a), carboxyl esterases from microbial communities inhabiting the shrimp *Rimicaris exoculata* dominating the fauna in deep-sea hydrothermal vent sites along the Mid-Atlantic Ridge (Alcaide et al., 2015b), an amylase from hydrothermal deep sea vents (Wang et al., 2011), and cellulolytic and hemicellulolytic enzymes from a naturally heated volcano site (Mientus et al., 2013).

In this study, we uncovered various hydrolase enzymes from diverse environments by functional screenings of mixed genomic DNA libraries from mesophilic, thermophilic, and hyperthermophilic microorganisms in the expression host *E. coli* at various temperatures. From 60 active clones, the majority (60%) of the enzymatic activities were observed when screening was performed at *E. coli*’s optimal growth temperature between 30 and 37°C, and a fraction of 40% was found to be active at lower or higher screening temperatures. Out of 20,000 single fosmid clones, one originating from a HA library screened at 70°C carried a particularly interesting fosmid encoding cellulolytic activity. Sequence analysis and subcloning experiments revealed that the gene responsible for this activity encoded a GHF12 endoglucanase termed Cel12E, which was characterized in more detail. The deduced Cel12E primary structure as well as the neighboring ORFs on the fosmid insert suggested that the gene originated from an extremely thermophilic archaeon possibly related to the genus *Thermococcus*.

A surprisingly low number of GHF12 proteins from hyperthermophilic archaea (*Sulfolobus solfataricus* P2, *Pyrococcus furiosus* DSM 3638, and *Caldivirga maquilingensis* IC-167) have been identified and characterized so far. They are all remarkably thermostable proteins with extremely high temperature optima between 80 and 100°C, while pH optima, substrate specificities, and activities can vary substantially (Bauer et al., 1999; Limauro et al., 2001; Huang et al., 2005; Girfoglio et al., 2012).

The GHF12 (formerly known as cellulase family H) belongs to glycoside hydrolase clan C, the members of which have a β-jelly roll structure with two glutamate residues serving as catalytic nucleophile/base and catalytic proton donor in a retaining mechanism of hydrolysis. According to the CAZy database^2^ (Lombard et al., 2014), GHF12 family proteins with endoglucanase activity (EC 3.2.1.4) are found in all domains of life, while β-1,3-1,4-glucanase (EC 3.2.1.73), xyloglucan hydrolase (EC 3.2.1.151), and xyloglucan endotransglycosylase (EC 2.4.1.207) activities seem mainly to be restricted to eukaryotes. The substrate spectrum of Cel12E determined by us confirmed the predicted endoglucanase activity, as it was able to hydrolyze mainly β-1,4-glycosidic cellulosic polysaccharides like CMC, β-glucan, hydroxyethylcellulose, and PASC, with only little activity on microcrystalline cellulose. It is interesting to note that Cel12E displays activity toward xyloglucan and xylans, which has not been previously reported for prokaryotic GH12 enzymes. The *in silico* characterization of the Cel12E protein revealed the presence of two CBMs at the C-terminus, which both belong to the CBM2 family. CBMs of this family can be divided into two types, based on the structural properties of the substrate they bind (Simpson et al., 2000). Cel12E was found to bind to cellulose but not to xylans. The presence of two CBMs can be explained as an adaptation to efficiently bind polysaccharides at extremely high temperatures. Tandem CBMs increase the affinity for polysaccharides by a factor of 10 to 100 compared to single CBMs, and since glycoside hydrolases with multiple CBMs occur most frequently in thermo- or hyperthermophilic organisms, CBM duplication may be a way to compensate for the loss of binding affinity that is observed with most molecular interactions at higher temperatures (Boraston et al., 2004).

Cel12E has a unique multidomain architecture that does not seem to exist in known proteins from other organisms. Other archaeal proteins comprising CBM2 domains seem to be exclusively connected to GH18 catalytic modules, as in the case of *P. furiosus* DSM3638 chitinase ChiA and ChiB (Oku and Ishikawa, 2006; Nakamura et al., 2008) and *Thermococcus kodakarensis* KOD1 ChiA (Tanaka et al., 1999). Interestingly, Cel12E also displayed chitin-binding ability, which presumably is brought about by its CBM2 modules, although no chitin-degrading activity was observed. Future experiments will help to clarify if the Cel12E CBM2 modules are responsible for the observed binding to cellulose, or if this capacity is due to other parts of the protein.

The supplementation with ions has been shown to specifically inhibit or enhance enzymatic activities observed in glycoside hydrolases from thermophilic organisms, although the mechanism is not well understood. For example, the presence of certain divalent metal ions was found to be essential for activity stimulation (Gargallo et al., 2006) and/or (thermal) stabilization of other enzymes (Morag et al., 1991; Abou-Hachem et al., 2002; Santos et al., 2012). In the case of the GH12 endocellulase EGPf of *P. furiosus*, crystallographic data and examination of thermostability showed a binding motif for divalent ions (Ca^2+^), which plays a functional role in thermostability (Kim et al., 2012). The mechanism of activation of CelE12 by low concentrations of manganese or cobalt ions remains to be elucidated.

Our data demonstrate that Cel12E is a cellulose-/β-glucan-specific endoglucanase, and based on sequence similarity of the neighboring ORFs found on the fosmid insert, we conclude that it originates from one of the uncharacterized hyperthermophilic archaea strains that were cultivated from deep sea vents. The enzyme may indicate the presence of certain β-glucan polysaccharides in the native environment, which are directly utilized by the organism, or which serve as storage polysaccharides. Another function of Cel12E may be in the metabolism of extracellular polysaccharides (EPS), which have been found in many marine organisms including hyperthermophilic archaea (Rinker and Kelly, 1996). EPS are high molecular weight carbohydrates that form complex heteropolysaccharides containing mainly mannose, glucose, galactose, and *N*-acetylglucosamin (Poli et al., 2011). EPS serve for cell attachment onto surfaces and protect the encapsulated cells from different types of environmental stress.

Although the physiological role of Cel12E remains unclear (its natural producer organism has not been characterized yet), its unique properties make this enzyme an interesting candidate for applications such as the degradation of cellulosic biomass under harsh reaction conditions.

## Conflict of Interest Statement

The authors declare that the research was conducted in the absence of any commercial or financial relationships that could be construed as a potential conflict of interest.

## Supplementary Material

The Supplementary Material for this article can be found online at http://journal.frontiersin.org/article/10.3389/fbioe.2015.00095

## Footnotes

^1^http://www.univ-brest.fr/souchotheque/Collection+LM2E

^2^http://www.cazy.org
