# Supplementary material for: Functional Screening of Hydrolytic Activities Reveals an Extremely Thermostable Cellulase from a Deep-Sea Archaeon
Source: Front Bioeng Biotechnol. 2015 Jul 1;3:95. doi: 10.3389/fbioe.2015.00095 (PMC4486847; doi:10.3389/fbioe.2015.00095)
Supplement: Supplementary file 2 [file table_2.docx]

**Supplementary Table S2**. Binding and effect of bivalent manganese and cobalt ions on Cel12E in the presence and absence of EDTA. Experiments were performed with 0.05 µg purified enzyme Cel12E against 2 % (w/v) carboxymethyl cellulose at 92 °C using 50 mM MES buffer (pH 5.5). Values are shown as relative activity values from duplicate measurements ± standard deviations.

|  |  |  |  |  |  | |  |
| --- | --- | --- | --- | --- | --- | --- | --- |
| Order of additive supplementation | EDTA | CoCl_2_ | MnCl_2_ |  | Relative activity (%) |  | |
|  |  |  |  |  |  |  | |
| No addition | - | - | - |  | 100.0 ± 9.2 |  | |
| 10 mM EDTA only | + | - | - |  | 88.5 ± 2.2 |  | |
| 1 mM CoCl_2_ only | - | + | - |  | 186.1 ± 2.5 |  | |
| 1 mM MnCl_2_ only | - | - | + |  | 178.4 ± 1.5 |  | |
| Addition of metal ions first: |  |  |  |  |  |  | |
| 1 mM CoCl_2_ + 10 mM EDTA | + | + | - |  | 107.7 ± 7.8 |  | |
| 1 mM MnCl_2_+ 10 mM EDTA | + | - | + |  | 82.9 ± 0.2 |  | |
| Addition of EDTA first: |  |  |  |  |  |  | |
| 10 mM EDTA + 1 mM CoCl_2_ | + | + | - |  | 102.2 ± 2.2 |  | |
| 10 mM EDTA + 1 mM MnCl_2_ | + | - | + |  | 106.9 ± 13.0 |  | |
|  |  |  |  |  |  |  | |
